# Ethnobotanical study and phytochemical profiling of *Heptapleurum hypoleucum* leaf extract and evaluation of its antimicrobial activities against diarrhea-causing bacteria

**DOI:** 10.1186/s43141-020-00030-0

**Published:** 2020-06-15

**Authors:** S. M. Rakib-Uz-Zaman, Asif Iqbal, Sadrina Afrin Mowna, Mst Gitika Khanom, Mohammad Mastak Al Amin, Kashmery Khan

**Affiliations:** 1 Biotechnology Program, Department of Mathematics and Natural Sciences, School of Sciences, BRAC University, Dhaka, Bangladesh; 2 Department of Biotechnology and Genetic Engineering, Islamic University of Kushtia, Kushtia, Bangladesh

**Keywords:** Ethnobotanical study, Phytochemical screening, Medicinal plants, Diarrhea, Herbal medicines, *Heptapleurum hypoleucum*, Antimicrobial activity, Antibiotic resistance

## Abstract

**Background:**

Due to the development of superbugs as a result of unprescribed and frequent use of antibiotics in recent years, an alternate form of medicine had to be introduced. In light of this global threat, researchers all over the world have been gravitating towards herbal medicines. In order to find out new ways of saving the planet using medicinal plants, ethnobotanical studies must be carried out. Concerning this, an ethnobotanical study has been done in this paper to identify potential medicinal plants in Rangamati, Bangladesh.

**Results:**

For the ethnobotanical survey, randomized 104 people were interviewed and 62 different plant species were found to treat 19 different kinds of diseases and 84% of people reported to be completely recovered. Furthermore, among the 19 diseases found, the majority of them were common cold, abdominal pain or gastric, diarrhea, and dysentery. From the 62 different plant species, *Heptapleurum hypoleucum*, used for the treatment of diarrhea, was selected for conducting further studies due to its heavy use as reported by the tribal people. In this study, the aqueous, ethanol, and methanol extracts of *Heptapleurum hypoleucum* were subjected to microbial susceptibility assays using the agar well diffusion method. The test microorganisms were *Salmonella typhi*, *Streptococcus pneumoniae*, *Staphylococcus aureus*, *Shigella flexneri*, *and Escherichia coli.* Among these, the most susceptible organisms were *Staphylococcus aureus* (21 mm) and *Salmonella typhi* (19 mm) in the ethanolic extract. Also, the methanolic extract showed an inhibition zone of 13 mm against *E. coli*, which was more than that of the antibiotic’s (11 mm). Phytochemical screening of the plant revealed that it contains alkaloids, phenols, steroids, and flavonoids, but lacks saponins and tannins.

**Conclusion:**

To combat the rising threat of antibiotic resistance, ethnoscience needs to be consolidated with modern biotechnological techniques to make the most use of the vast amount of natural resources. The findings of this study indicate that *Heptapleurum hypoleucum*, an ethnobotanical medicinal plant, has shown comparable antimicrobial activity with commercial antibiotics against several diarrhea-causing pathogens and also contains several medically important phytochemicals.

## Background

The discovery of antibiotics has been a blessing to the modern world. Sadly, the uncontrolled and unprescribed use of antibiotics has turned them into the enemy of human existence as microorganisms are becoming multi-drug resistant. Given this, researchers and scientists agree that medicinal plants are a substantial alternative to this global crisis [1]. However, to discover new species of medicinally important plants, ethnobotanical surveys are needed to be conducted. In respect to that, an ethnobotanical study has been done as a part of this research paper to identify some of the plants used by tribal people in Rangamati, Bangladesh.

Medicinal plants are a common phenomenon in various continents and are also an essential source of different medicines [2]. Being a developing country, the use of medicinal plants in Bangladesh is notable in many regions. Therefore, medicinal plants have become one of the leading choices for treating different injuries and diseases. A significant portion of the plant species has been recognized as valuable resources of natural antimicrobial compounds which can be used as an alternative for the treatment of antibiotic-resistant bacterial infections [3]. They are the most suitable solution to the problems regarding antibiotic resistance, as they are cost-effective and have the least amount of side effects [4]. Medicinal plants exhibit antibacterial activities because of the phytochemicals produced during the secondary metabolism of the plants [5–7]. Plants are rich in a wide variety of secondary metabolites, including tannins, alkaloids, phenolic compounds, and flavonoids [8, 9]. These phytochemicals are known to exhibit anti-inflammation, antimicrobial, and antifungal activities [5]. Reports suggest that phenolic and flavonoid compounds prevent diseases such as cancer, heart problems, cataracts, eye disorders, and Alzheimers [6]. The most vital properties of flavonoids include their ability to protect against oxidative diseases, reduce the oxidation of low-density lipoproteins to provide protection against cardiovascular diseases, and activate or inhibit various enzymes bind specific receptors [10].

Moreover, it is suggested that herbal extracts may be an effective alternative to antibiotics in order to cure common recurrent diseases like diarrhea, skin disease, throat, or ear infection [11]. Surprisingly, medicinal plants have also shown their efficiency in treating diabetes due to the presence of carotenoids, flavonoids, terpenoids, alkaloids, and glycosides [12]. The anti-hyperglycemic effects that result from treatment with plants are often due to their ability to improve the performance of pancreatic tissue, which is done by increasing insulin secretions or reducing the intestinal absorption of glucose [13].

A survey conducted among the Santal tribe of Joypurhat District, Bangladesh, found 33 plant species that were used by the tribal people for treating diabetes (A.H.M [14]).

Ethnobotanical surveys in our country [15, 16] have brought plants having effective medicinal characteristics into attention. Some studies have been done specifically to search for different medicinal plants, which are being used for the treatment of diarrhea and dysentery [17, 18]. After conducting the survey, a medicinal plant—*Heptapleurum hypoleucum*, locally known as Jharobbo hogoeya (Bangladesh Ethnobotany Online Database), was collected to evaluate its antibacterial activity against some pathogenic microorganisms responsible for causing diarrhea. This plant grows in the forest regions of the Rangamati, and people from the survey have claimed to use this plant for the treatment of diarrhea and dysentery. Tribal people are extremely experienced as they have been treating diseases using medicinal plants since prehistoric times and thus are knowledgeable about herbal medicine including those used for the treatment of diarrhea (M.S. [16]).

In this study, the test microorganisms were *Salmonella typhi*, *Streptococcus pneumoniae*, *Staphylococcus aureus*, *Shigella flexneri*, and *Escherichia coli*. The aqueous, ethanol, and methanol extracts of *Heptapleurum hypoleucum* were subjected to microbial susceptibility assays using the agar well diffusion method. After the phytochemical screening of plant extract, we have successfully detected alkaloids, phenolics, steroids, and flavonoids in different solvents. However, no saponins and tannins were identified in our extract. This study reveals the antibacterial effects of ethnobotanical medicinal plants as well as the presence of phytochemicals that may have antimicrobial activity against diarrhea-causing pathogenic microorganisms.

## Methods

### Survey site selection

The region selected for this research purpose has an area of 6116.13 km^2^ and is located in between 22°27′ and 23°44′ north latitudes and in between 91°56′ and 92°33′ east longitudes. Approximately 5,08,182 people live here and the main indigenous communities that live here are Chakma, Monipuri, Tripura, Khumi, Marma, Tanchangya, Santal, Mro, and many more.

### Ethics and consent to participate

The purpose of our study was elaborately explained to each informant and verbal consent was taken to avoid any misunderstanding. We have also taken permission from the local authority to conduct the survey. As our study only dealt with the medicinal plants with no intention to conduct any trials on human, formal institutional consent for this study is not required. In addition, Bangladesh Medical Research Council Ethical Guidelines for Conducting Research Studies Involving Human Subjects deemed ethics approval unnecessary for this kind of study on page 39, section 10.5.2.

### Data analysis

All the data were listed alphabetically and ordered by the plant’s scientific name, local name, plant part used, name of the disease, and mode of preparation of the plant. Also, the data were analyzed in the “IBM SPSS Statistics 25” software and graphical presentations were made.

### Antibacterial activity test

#### Test organisms

The pathogenic test organisms used in this assay are as follows: *Salmonella typhi*, *Streptococcus pneumoniae*, *Staphylococcus aureus*, *Shigella flexneri*, and *Escherichia coli*, all are known diarrhea-causing bacteria. The bacterial cultures were obtained from the Biotechnology and Molecular Biology Laboratory of the Department of Mathematics and Natural Sciences at BRAC University and the International Centre for Diarrheal Disease Research, Bangladesh (ICDDR, B).

#### Sample collection and processing

With the help of some local practitioners, parts of the *Heptapleurum hypoleucum* plant were taken and identified. The stem of the plant was used as the sample for this research work, obtained from the rural areas of Rangamati district. The stem was washed, cleaned, and air dried under open light for several days. As soon as it dried, it was mashed into powder.

### Preparation of extracts using different solvents

#### Ethanolic extraction

Ten grams of powdered sample was dissolved in 100 ml of absolute ethanol in a conical flask, covered with aluminum foil and then kept at 37 °C in a shaker incubator at 120 rpm for 24 h. Using an autoclaved filter paper, the filtrate was collected slowly in a conical flask and then evaporated using a rotary evaporator till the final volume was reduced to one-fourth of the original volume of the solvent used. This concentrated extract solution was poured on a sterile petri dish lid and kept in the incubator at 55 °C for 20 min. Finally, a sticky semi-solid extract appeared on the surface of the plate when all the solvent was evaporated. The extract was collected and stored in a McCartney bottle that was autoclaved and weighed. The extract inside the bottle was weighed, recorded, and the exact amount of the extract was calculated by subtracting the mass of the empty bottle. Then the bottle was labeled and stored at 4 °C in the refrigerator.

#### Methanolic extraction

Ten grams of powdered sample was dissolved in 100 ml of absolute methanol in a conical flask, covered with aluminum foil and then kept at 37 °C in a shaker incubator at 120 rpm for 24 h. The filtrate was collected slowly in a conical flask using an autoclaved filter paper. It was then evaporated using a rotary evaporator till the final volume was reduced to one-fourth of the original volume of the solvent used. Then the concentrated extract solution was poured on a sterile petri dish lid and kept in the incubator at 55 °C for 20 min. Finally, a sticky semi-solid extract appeared on the surface of the plate when all the solvent was evaporated. The extract was collected and stored in a McCartney bottle that was autoclaved and weighed. The extract inside the container was weighed, recorded, and the exact amount of the extract was calculated by subtracting the mass of the empty bottle. Then the bottle was labeled and stored at 4 °C in the refrigerator.

#### Aqueous extraction

Ten grams of powdered sample was weighed and mixed with 100 ml of distilled water in a conical flask, covered with aluminum foil. Then kept at 37 °C in a shaker incubator at 120 rpm for 24 h. Using an autoclaved Whatman No.1 filter paper, the filtrate was collected slowly in a conical flask and then stored inside autoclaved falcon tubes at 4 °C in the refrigerator.

### Preparation of extract solution for antibacterial activity test

The following formula was used to determine the amount of solvent to be added for making the extract solution for antibacterial activity test.

$$ \mathrm{Amount}\ \mathrm{of}\ \mathrm{solvent}\ \mathrm{to}\ \mathrm{be}\ \mathrm{added}=\frac{100\times \mathrm{amount}\ \mathrm{of}\ \mathrm{extract}\ \mathrm{obtained}}{\mathrm{amount}\ \mathrm{of}\ \mathrm{plant}\ \mathrm{powder}\ \mathrm{used}} $$

#### Agar well diffusion

Antibacterial activity of aqueous and solvent extracts was determined based on agar well diffusion method developed by Clinical and Laboratory Standards Institute, USA with some modifications depending on our laboratory conditions [19].

Plates of bacterial strains were taken in the laminar hood. A loop was sterilized in the Bunsen flame and was used to scrape off the bacteria and dipped into the test tubes containing saline solution (each containing 9 ml of NaCl) to make a suspension. The test tubes were vortexed and the turbidity of the suspension was visually compared with the 0.5% MacFarland standard solution in order to keep the number of bacteria in the saline suspension within a given range for standardizing the lawn culture of antimicrobial tests. Then, an autoclaved cotton swab was dipped into the suspension and pressed against the inner walls of test tubes to remove excess liquid before taking them out. The cotton swab was rubbed horizontally across the surface of the labeled Mueller Hinton Agar (MHA) plates to conduct the lawn culture of the bacterial strains. A cork borer was heated to sterilize, cooled, and then pressed onto the MHA plates to create the required number of wells on the quadrants of the agar. After that, each well was labeled and filled with 60 μL of diluted methanolic, ethanolic, and aqueous extracts, respectively. Different antibiotic disks were used as a positive control and placed onto one quadrant. Then the MHA plates were kept in the incubator for 24 h at 37 °C and the results were observed and recorded the next day.

All the tests were conducted 3 times to obtain the average value of zones of inhibition. The zones were measured using a millimeter scale and then the activity index for each extract was calculated.

The following formula was used for calculating the activity index:

$$ \mathrm{Activity}\ \mathrm{index}=\frac{\mathrm{zone}\ \mathrm{of}\ \mathrm{inhibition}\ \mathrm{of}\ \mathrm{plant}\ \mathrm{extract}}{\mathrm{zone}\ \mathrm{of}\ \mathrm{inhibition}\ \mathrm{of}\ \mathrm{antibiotic}\ \mathrm{disc}} $$

### Biochemical assays and phytochemical analysis

The plant extracts in methanolic, ethanolic, and aqueous solutions were assessed for the existence of the phytochemical compounds based on the following standard methods [20–23].

#### Tests for alkaloids

The methanolic extract was diluted in an acidic solution of HCl. This test solution was used for the detection of alkaloids using various reagents.

Hager’s test: 1 ml of extract was carefully mixed with 3 drops of freshly prepared Hager’s reagent in a test tube. The formation of yellow precipitates represents a positive result and the presence of alkaloids in the extract.

Wagner’s test: 1 ml of extract was mixed in a test tube with 3 drops of Wagner’s reagent prepared beforehand. The formation of brown precipitate will indicate the presence of alkaloids.

Dragendraff’s test: 2 ml of extract was taken in a test tube with 0.2 ml diluted HCl and 1 ml of Dragendraff’s reagent and left for a few minutes. A positive result is indicated by the presence of an orange-brown precipitate.

#### Test for steroidal compounds

Salkowaski’s test: 0.5 g of the extract was dissolved in 2 ml chloroform in a test tube. Concentrated sulfuric acid was carefully added on the wall of the test tube to form a lower layer. A reddish-brown color at the interface will indicate the presence of a steroid ring.

#### Test for phenolic compounds

Equal amounts of 1% ferric chloride solution and 1% potassium ferrocyanide were mixed. Then, 3 drops of this freshly prepared mixture were added to 2 ml extract. The formation of a bluish-green color will represent a positive result.

#### Test for flavonoids

Reaction with sodium hydroxide: 2 ml diluted NaOH solution was added to 3 ml of extract. The mixture was inspected for the production of yellow color, which is considered positive.

#### Tests for saponins

Froth test: 0.5 g of the extract was dissolved in 10 ml distilled water. The test tube was stoppered and then shaken vigorously for 30 s. It was then allowed to stand for 30 min. Formation and retention of honey-comb froth on the surface for 30 min is considered positive for saponins.

#### Tests for tannins

Lead acetate test: 5 ml of extract and a few drops of freshly prepared 1% lead acetate were dissolved together. The formation of a yellow precipitate is considered a positive result.

## Results

### Ethnobotanical survey

In this survey, it was found that 101 people out of 104 have used or been using medicinal plants. Eighty-five people reported to be fully recovered using the respective medicinal plants, 15 people recovered partially, and 7 people faced some side effects during their treatment. From Table 1, we can see that the percentage of people using medicinal plants was the highest (30.77%) for the age group 30–40 and the percentage of people aged above 60 was the least (6.73%). The second-highest percentage (26.92%) of people using plants as medicine was for the age group 51–60, while the second least percentage (11.54%) of people using herbs was aged less than 30. Medicinal plants were used more by men (74.04%) than women (25.96%). Most people using herbal medicines had a job or a business (20.19% both) and the second-highest percentage of people using medicinal plants were housewives (17.31%). Farmers (14.42%) and workers (11.54%) seemed to use herbal medicines more than unemployed people (8.65%), although, still less than housewives. Lastly, the lowest number of people (1.92%) utilizing herbs were guide by profession (Table 1).

**Table 1:** Demographic data of the informants. The percentage of people using medicinal plants was highest (30.77%) for the age group 30–40 and the percentage of people aged above 60 was the least (6.73%). In addition, considering the profession, students (5.77%) and guides (1.92%) used the least amount of herbal medicine, while local businessmen and job holders used the highest amount (both 20.19%) of medicinal plants to treat different diseases and illness

Variable	Categories	Frequency (*n* = 104)	Percentage (%)
Age	<30 30–40 41–50 51–60 Above 60	12 32 25 28 7	11.54 30.77 24.04 26.92 6.73
Gender	Male Female	77 27	74.04 25.96
Profession	Worker Job Housewife Guide Business Farmer Unemployed Student	12 21 18 2 21 15 9 6	11.54 20.19 17.31 1.92 20.19 14.42 8.65 5.77

**Fig. 1:**
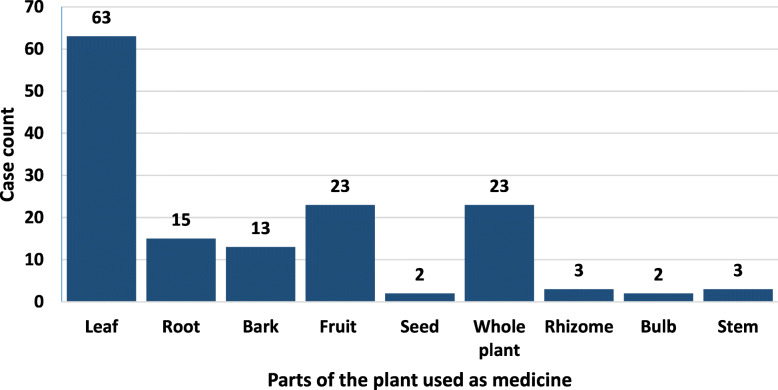
Part of the plant used as medicines. The most used part of the plant was leaves (42.86%) and the least used parts were the seed and bulb (1.36%)

In this survey, 42.86% of the plant part used were the leaves, confirming the fact that leaves are the most used part of a plant (Fig. 1). The second most used parts were fruits and whole plants, each having 15.65% of the total count. The least used parts were the seed and bulb, having just 1.36% each. The second least used parts were the rhizome and stem, being only 2.04% each. The percentage of bark used (8.84%) can be termed as the third least part used as it has a higher percentage of use than the seed, rhizome, bulb, and stem. The root (10.20%) is utilized more than the bark but still less than the fruit, whole plant, and leaves (Fig. 1).

**Fig. 2:**
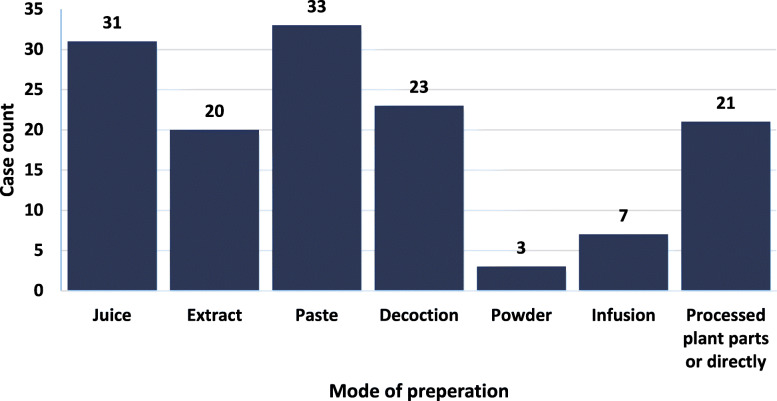
Mode of preparation of plant parts. The most used form of preparation was “paste” (23.91%) and the least used was “powder” (2.17%)

In this survey, 33 people preferred to make “paste” from the plant parts (Fig. 2), which is also the highest count and shows the highest percentage (23.91%). From Fig. 2, we can see that plant parts were also taken in the form of “juice” by 31 people, which is the second-highest form (22.46%). In addition, 23 and 20 people used the “decoction” (16.67%) and “extraction” (14.49%) method respectively. Twenty-one people took the whole plant as medicine or processed in some way like cooking (15.22%), which is lower than for the decoction method and higher than for the extraction method. The lowest number of people, 3, took the plant in a powdered form (2.17%) and the second-lowest number of people, 7, used the plant as an infusion (5.07%). In summary, the highest percentage of people used medicinal plants in the form of “paste,” while the least number of people used the plant in the “powdered” form.

**Fig. 3:**
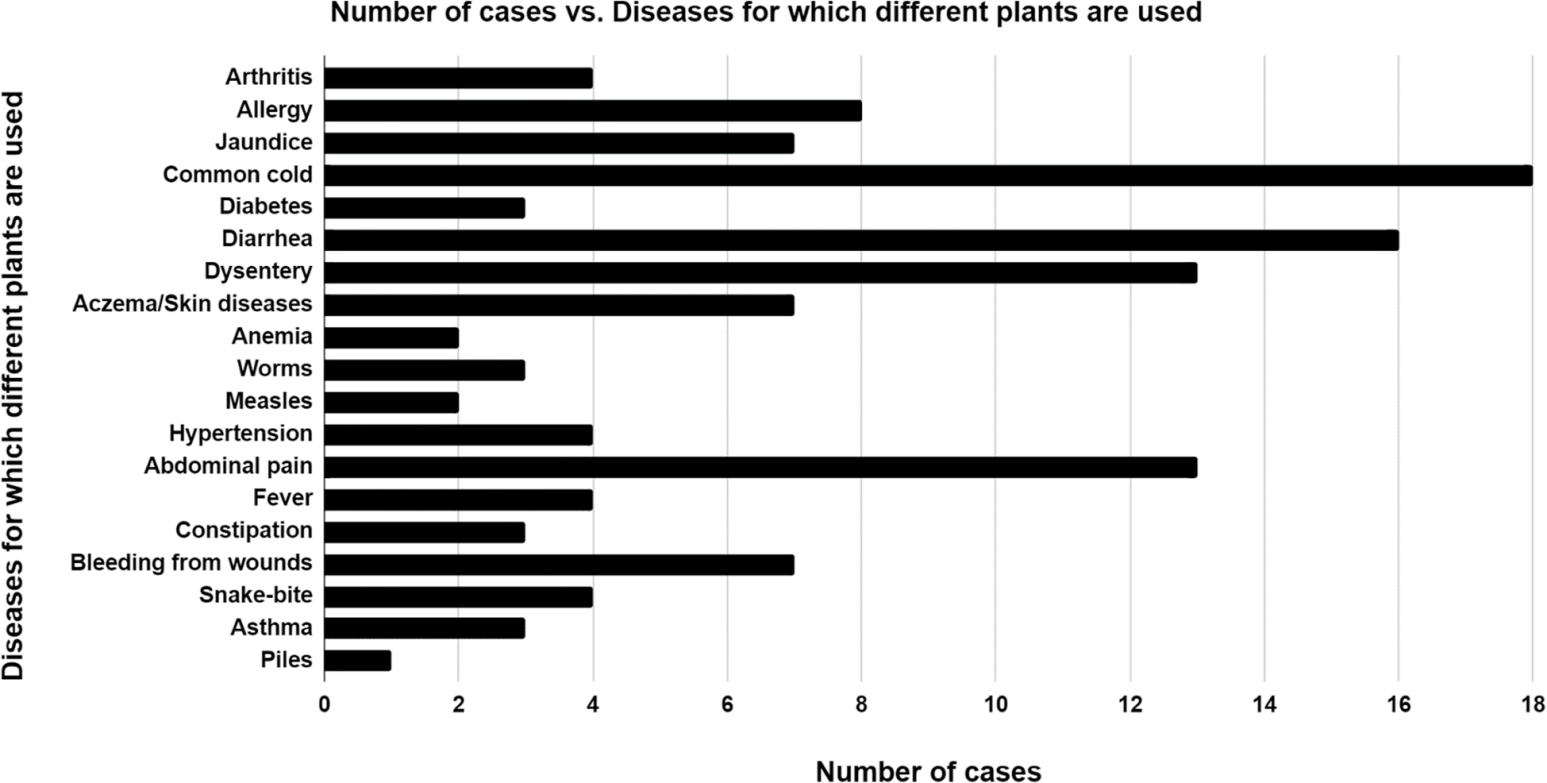
Diseases for which different plants are used. The highest count of occurrence was for common cold, while the least was for piles

A wide range of diseases was cured using different kinds of plants. The diseases include minor injuries, common viral infections, and also bacterial diseases, all common throughout our country, Bangladesh. In this survey, common cold, abdominal pain, diarrhea, dysentery, and allergy or rashes are the most common ones, including 19 types of different diseases in total. From Fig. 3, we can conclude that anemia, piles, and measles were the least common. The highest count of occurrence was for common cold, while the second-highest count was for diarrhea among the participants of this study. The least count of occurrence was for piles and the second least count was for both anemia and measles. Although the count of occurrence was quite high for dysentery and abdominal pain and gastric, it was still lower than for common cold, throat pain, and diarrhea. The cases of arthritis, diabetes, worms, hypertension, fever/dengue, constipation, snake-bite, and asthma were very low, but they were still higher than the occurrence of piles, allergy, jaundice, eczema/skin disease, and bleeding from wounds (Fig. 3).

### Ethnomedicinal plants used by the tribal people of Rangamati

A total of 62 medicinal plants were found to treat several diseases (Table 2). Out of the 26 plants treating gastrointestinal ailments (GIA), some plants are *Azadirachta indica*, *Canna indica*, and *Heptapleurum hypoleucum.* Similarly, 12 plants including *Allium sativum* were found to treat respiratory system disorders (RSD). Poisonous bites (PB) and skeleton muscular system disorders (SMSD) were treated by 2 plants, including *Alocasia cucullate* and *Brassica nigra* respectively. Endocrinal disorders (ED), throat pain, and hemorrhoids (HEM) were healed by *Andrographis paniculate*, *Leea macrophylla*, and *Nelumbo nucifera* respectively. Four plants, together with *Stephania japonica*, were used as a remedy for different types of fever (Fvr)*.* To treat liver problems (LP), five plants were used, including *Carica papaya* and *Hibiscus sabdariffa.* Dermatological infections/diseases (DID) were treated using nine different plants, like, *Bridelia retusa*, *Cassia alata*, and *Curcuma longa.* Three plants were used to treat hypertension (HTN) and two were used to treat Anemia, along with *Stephania japonica* and *Basella alba* respectively (Table 2). *Heptapleurum hypoleucum* was the most frequently used plant for the treatment of diarrhea as reported by the participant of this study which motivated us to choose this plant to further investigate its antimicrobial potentials against diarrhea-causing pathogens.

**Table 2:** Ethnomedicinal plants used by the tribal people of Rangamati for treating different diseases (source: Medicinal Plants Database of Bangladesh [24–26]). A total of 62 medicinal plant species were found out of which 26 plants treat gastrointestinal ailments (GIA), 12 plants treat respiratory system disorders (RSD). Poisonous bites (PB) and skeleton muscular system disorders (SMSD) were treated by 2 plants. Endocrinal disorders (ED), throat pain, and hemorrhoids (HEM) were healed by 3 plants. Four plants were used to cure different types of fever (Fvr). For liver problems (LP), 5 plants were used. Dermatological infections/diseases (DID) were treated using 9 different plants. Three plants were used to treat hypertension (HTN) and 2 were used to treat anemia

Botanical/scientific name of the plant	Local name	Illness/disease treated	Part of the plant used	Mode of preparation
*Aegle marmelos*	Bel	Abdominal pain, diarrhea	Fruit	Juice
*Allium sativum*	Roshun	Common cold	Bulb	Juice
*Alocasia cucullate*	Bilai kochu	Snake bite	Leaves	Paste
*Aloe Barbadensis*	Ghritokumari	Constipation	Whole plant	Juice
*Andrographis paniculate*	Kalomegh	Diabetes	Whole plant	Juice
*Anisomeles indica*	Jongli horinchi	Child fever	Leaves	Juice
*Artocarpus heterophyllus*	Kathal	Diarrhea	Leaves	Decoction
*Averrhoa carambola*	Kamranga	Jaundice	Fruit	Whole fruit
*Azadirachta indica*	Neem	Allergy	Leaves	Paste and juice
*Basella alba*	Pui Shaak	Anemia	Whole plant	Processed plant
*Brassica nigra*	Sorisha	Arthritis	Leaves	Extract
*Bridelia retusa*	Shukujjaa	Eczema	Leaf	Paste
*Canna indica*	Kolaboti	Intestinal worms	Rhizome	Juice
*Carica papaya*	Pepe	Diarrhea, jaundice	Fruit	Processed plant
*Cassia alata*	Dilong	Eczema	Leaves	Paste
*Catharanthus roseus*	Badam boot	Hypertension	Leaf	Juice
*Centella asiatica*	Thankuni	Abdominal pain	Whole plant	Juice
*Cocos nucifera*	Narikel	Jaundice	Fruit	Juice
*Commelina paludosa*	Baat boitta shaak	Dysentery/diarrhea	Whole plant	Processed plant
*Costus speciosus*	Ketoki	Bleeding from wounds, constipation	Root	Extract
*Curcuma longa*	Holud	Eczema	Root	Paste
*Cymbopogon citratus*	Dhan-shabarang	Common cold	Leaves	Extract
*Cymbopogon citratus*	Leangra Gach	Snake bite	Root	Paste
*Cynodon dactylon*	Durbaghash	Bleeding from wounds	Whole plant	Paste
*Dryopteris filix-mas*	Dheki Shak	Common cold	Whole plant	Processed plant
*Eichhornia crassipes*	Kochuripana	Asthma, allergy	Leaves	Juice
*Glinus oppositifolius*	Gima shak	Allergy	Whole plant	Processed plant
*Gmelina arborea*	Gamari	Abdominal pain	Root	Decoction
*Heptapleurum hypoleucum*	Jharobbo hogeya	Diarrhea	Stem & Root	Decoction
*Hibiscus sabdariffa*	Amilla	Jaundice	Leaves	Decoction
*Holarrhena antidysenterica*	Kurchi	Asthma	Root	Extract
*Hordeum vulgare*	Barley	Diarrhea	Whole plant	Powder
*Jatropha gossypifolia*	Titto long	Dengue fever	Seed	Powder
*Justicia adhatoda*	Bashok	Common cold	Leaves	Juice
*Laurus nobilis*	Tej pata	Eczema	Leaves	Infusion
*Leea macrophylla*	Ash	Throat pain	Leaves	Juice
*Litsea monopetala*	Shurjo Pata	Diarrhea	Leaves	Decoction
*Mikania micrantha*	Ashamlata	Bleeding from wounds	Leaves	Paste
*Momordica charantia*	Korolla	Measles	Fruit	Processed plant
*Musa paradisiaca*	Kacha-kola	Blood dysentery	Fruit	Processed plant
*Musa sapientum*	Chompa-Kola	Blood dysentery	Fruit	Whole fruit
*Nelumbo nucifera*	Roktopoddo	Piles	Root	Extract
*Ocimum tenuiflorum*	Tulsi	Common cold	Leaves	Juice
*Pandanus odoratissimus*	Keorakata	Abdominal pain	Root	Paste
*Phyllanthus emblica*	Amloki	Common cold	Fruit	Fruit directly
*Piper betel*	Paan pata	Gastric	Leaves	Whole leaf
*Plumbago indica*	Agunitita	Blood dysentery	Leaf	Paste
*Psidium guajava*	Peyara	Blood dysentery	Leaves	Paste
*Punica granatum*	Bedana	Anemia	Fruit	Fruit directly
*Rourea minor*	Kurochik-shak	Diarrhea	Leaf	Infusion
*Saccharum arundinaceum*	Teng	Dysentery	Leaves	Decoction
*Saccharum officinarum*	Aakh	Jaundice	Fruit	Juice
*Senna alata*	Dao long	Ring worm	Leaves	Paste
*Stephania japonica*	Thandamanik gach	Fever, headache, hypertension	Leaves	Juice
*Swertia chirayita*	Chirota	Abdominal pain	Fruit	Processed plant
*Syzygium cumini*	Kalojaam	Dysentery	Fruit	Whole fruit
*Tamarindus indica*	Tetul	Hypertension	Fruit	Whole fruit
*Terminalia arjuna*	Arjun	Allergy	Bark	Decoction
*Urena lobate*	Barokra	Abdominal pain	Stem	Decoction
*Vitis sp.*	Khoijong	Fever, common cold	Root	Juice
*Wedelia trilobata*	Khetranga	Dysentery	Rhizome	Extract
*Zingiber officinale*	Aada	Abdominal pain, common cold	Rhizome	Juice

### Antibacterial assay

**Fig. 4:**
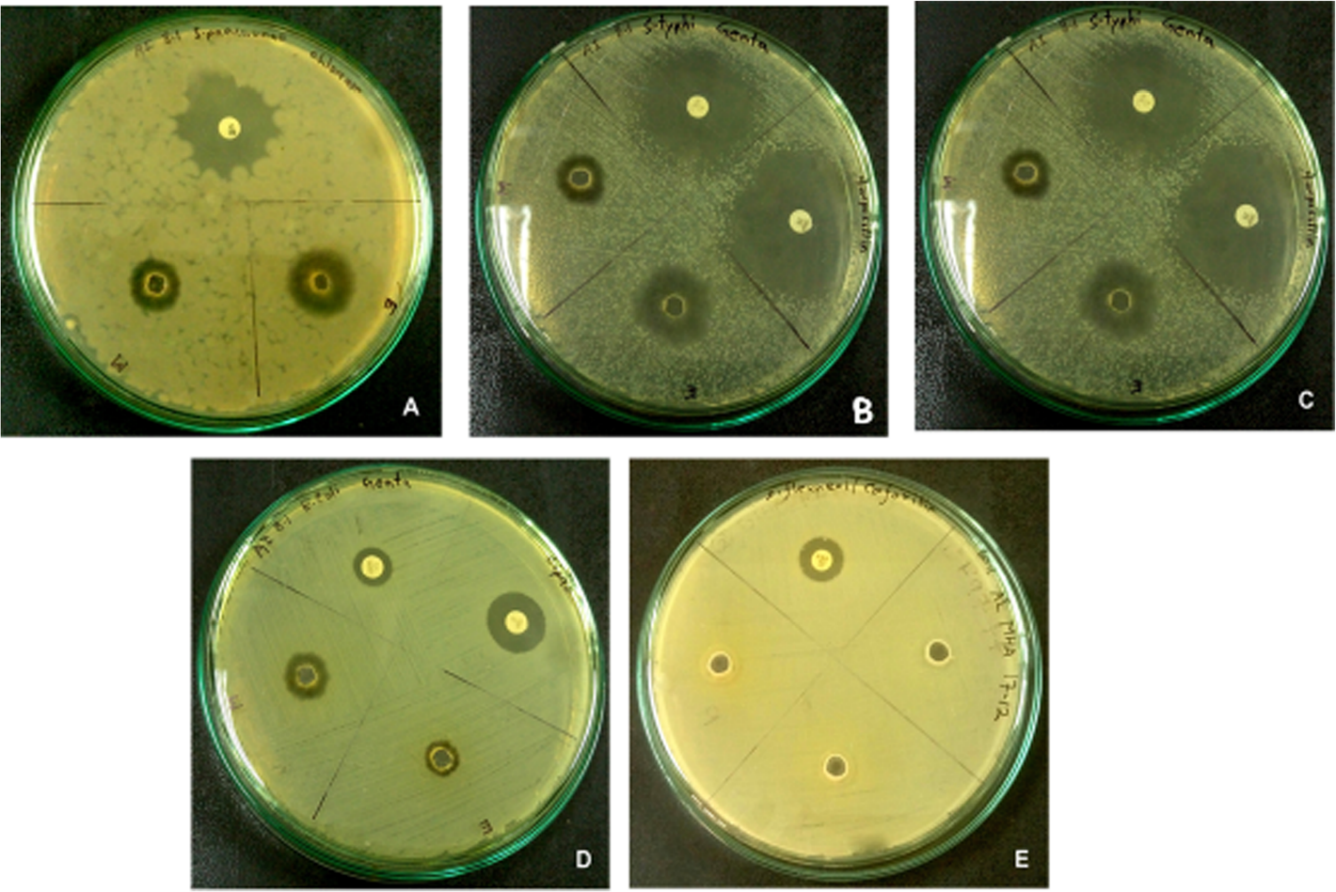
Antibacterial effects of ethanolic and methanolic extracts of *Heptapleurum hypoleucum* against. **a***S. pneumonae.***b***S. aureus*. **c***S. typhii*. **d***E.* coli. **e***Shigella flexneri.* Ethanolic extract showed the highest zone of inhibition against *Staphylococcus aureus* (21 mm). Each of the plates is also showing the zone (white) produced by the commercial antibiotics against respective bacteria (as mentioned in Table 3)

**Fig. 5:**
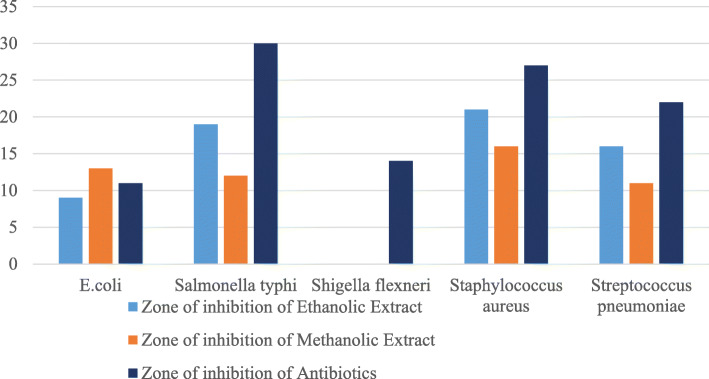
Graphical representation zone of inhibition (mm) of different extracts of *Heptapleurum hypoleucum* in comparison with antibiotic disks against some disease-causing microorganisms. Ethanolic extract showed the highest zone of inhibition against *Staphylococcus aureus* (21 mm)

The antibiotic disks used were ampicillin and gentamicin (for *S. typhii* and *S. aureus*), gentamycin (for *E.* coli), chloramphenicol (for *S. pneumonae*), and cefoxitin (for *S. flexneri*) (Fig. 4). The tests were repeated thrice to ensure the accuracy of the results. Among the three different types of extracts, the distilled water extract showed hardly any positive result. However, ethanolic and methanolic extracts were able to show some positive results, but not against all the organisms (Fig. 5). Ethanolic extract showed the highest zone of inhibition against *Staphylococcus aureus*, which was 21 mm (Table 3). The average result of the three trials are given below (Fig. 5).

**Table 3:** Antibacterial test results of the ethanolic, methanolic, and aqueous extracts of *Heptapleurum hypoleucum.* Ethanolic extract showed the highest zone of inhibition against *Staphylococcus aureus* (21 mm)

Extract (quadrant)	*Escherichia coli*	*Salmonella typhi*	*Shigella flexneri*	*Staphylococcus aureus*	*Streptococcus pneumoniae*
	Antibiotic used-Gentamicin	Antibiotic used-Gentamicin	Antibiotic used-Cefoxitin	Antibiotic used-Gentamicin	Antibiotic used-Chloramphenicol
The average diameter of zone of inhibition (mm) of antibiotics and different extracts/quadrant					
Antibiotic	11	30	14	27	22
Ethanol	9	19	0	21	16
Methanol	13	12	0	16	11
Distilled water	0	0	0	11	6
Activity index/quadrant					
Ethanol	0.81	0.63	0	0.78	0.73
Methanol	1.18	0.4	0	0.59	0.5

**Fig. 6:**
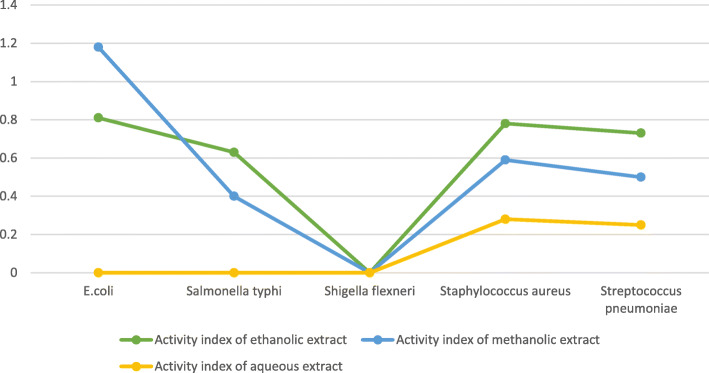
Activity index of ethanolic, methanolic, and aqueous extracts against different microorganism. *E. coli* had the highest activity index in the ethanolic extract (value 0.81) as well as in the methanolic extract (value 1.18), while the lowest was seen for *Shigella flexneri* (value 0 in all solvents)

The activity index of *E. coli* was the highest in the ethanolic extract (value 0.81) as well as in the methanolic extract (value 1.18), while the lowest was seen for *Shigella flexneri* (value 0 for all solvents) (Fig. 6). The activity index for aqueous extract was 0 for *E. coli*, *Salmonella typhi*, and *Shigella flexneri* but had a value of 0.28 for *Staphylococcus aureus* and a value of 0.25 for *Streptococcus pneumoniae*. Here, the activity index of ethanolic extract for *Streptococcus pneumoniae* (0.73) is seen to be lower than for *Staphylococcus aureus* (0.78) and higher than *Salmonella typhi* (0.63) (Fig. 6).

### Summary of the phytochemical screening

**Fig. 7:**
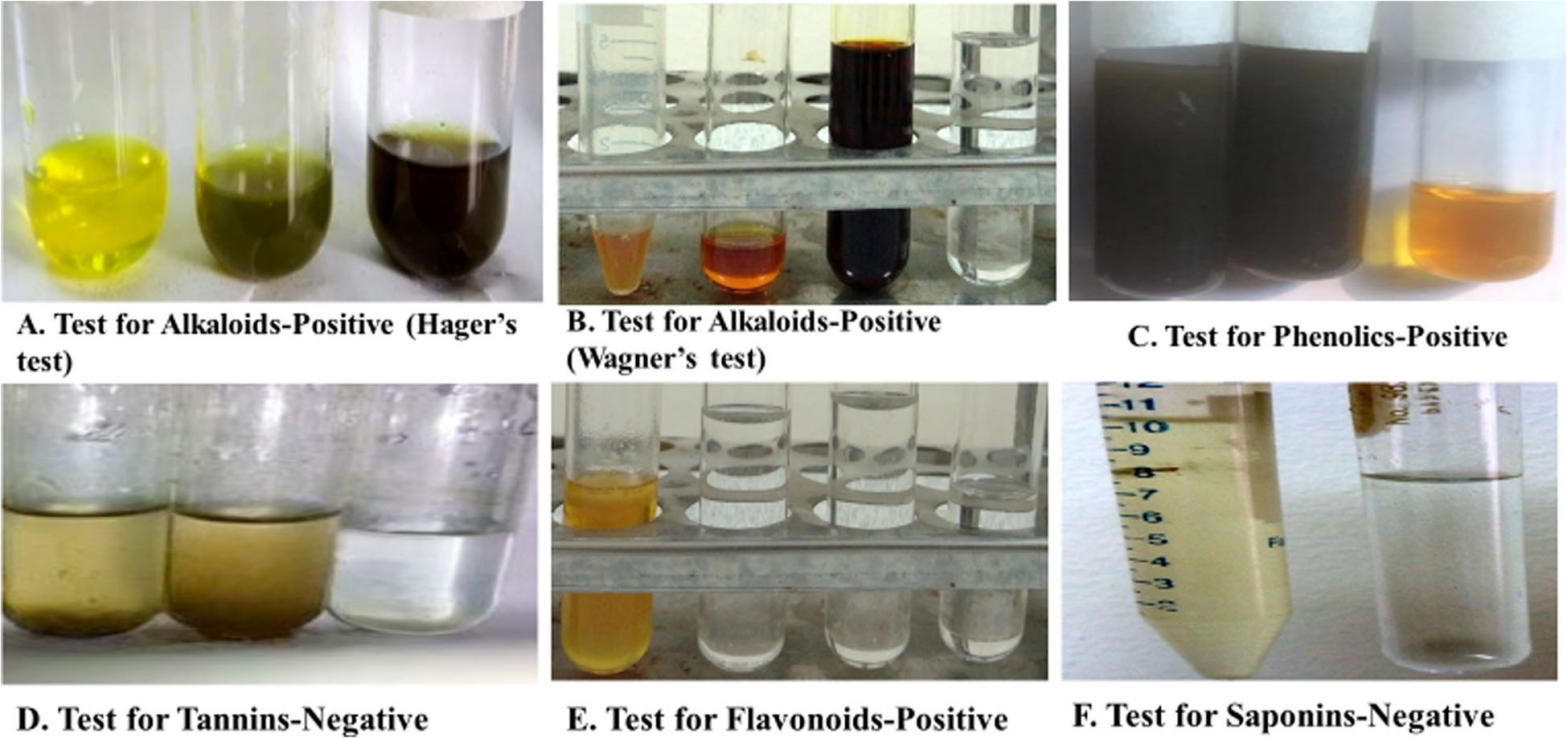
Figures are showing phytochemical test results for different plant compounds. **a** Alkaloids (Hager’s test): from left to right—Hager’s reagent, test result, and ethanolic extract. There was a visible color change of the sample which indicates a positive result for alkaloids. **b** Alkaloids (Wagner’s test): from the left side is the test sample, Wagner’s reagent mixture, Wagner’s reagent, and ethanol. The visible color change of the sample indicates a positive result for alkaloids. **c** Test for the identification of phenolic compounds. From the left side, the first two are sample extracts showing the desired color change which indicate positive result and the 3rd one is the control, FeCl_3_. A dark green color indicates the presence of phenolic compounds in the sample. **d** Tests for tannins (lead acetate test): no yellow precipitation in the test sample indicates a negative result. **e** Test for flavonoids: from the left side—1st one is test sample showing yellow color, 2nd one ethanol, 3rd one NaOH, and 4th one is the mixture of ethanol and NaOH. There was a visible color change of the sample which indicates a positive result for flavonoids. **f** Tests for saponins (froth test): the left one is the test sample and the other one is distilled water. No froth formed in the test sample indicates a negative result for saponins

We have performed 6 tests for screening the phytochemical compounds in our extract. The tests are for alkaloids, phenolic compounds, flavonoids, saponins, steroids, and tannins. We got positive results for alkaloids, phenolics, flavonoids, and steroids. However, negative results were observed for saponins and tannins (Fig. 7). The result of the phytochemical screening has been summarized in Table 4.

**Table 4:** Phytochemical analysis showing positive results for alkaloids, flavonoids, and steroids. In contrast, our study also indicates the absence of saponins and tannins in the sample

Name of the phytochemical	Result
Alkaloids	Positive
Phenolics	Positive
Flavonoids	Positive
Saponins	Negative
Steroids	Positive
Tannins	Negative

## Discussion

Tribal people have been using plants to treat different kinds of diseases for a long time. Years of experience has made them a “knowledge house” on herbal remedies. An ethnobotanical survey of medicinal plants in the Chittagong Hill Tracts has revealed that 56 plant species were used by the tribal people only to treat dermatological disorders [27]. In another ethnobotanical study, conducted among Pangkhua community in Bilaichari, Bangladesh, revealed the traditional use of 117 plant species to treat 11 categories of ailments, recorded from 218 traditional healers and elderly men and women [28]. Our study also found that 62 plant species were used by the tribal people of Rangamati to treat almost 20 different diseases, namely abdominal pain, common cold and sneezing, injury, diarrhea, and dysentery. In this study, about 77% of the participants reported that they had recovered from the above-mentioned diseases within 1 to 10 days using only the medicinal plants as a remedy. In contrast, only 2–3% of the participants took additional medications (besides the medicinal plants) which might contribute to their quick recovery. Our study also found that age has significant influence when choosing medicinal plants over traditional medications. Older people naturally ought to have a better understanding and greater knowledge about the traditional use of medicinal plants as pharmaceuticals or modern medicines were unavailable in their era. The fact of having superior knowledge about the use of medicinal plants is reflected to some extent in this survey as about 34% of the total people questioned aged above 50 years old. While using plants as remedies against diseases, leaves (42.68%) were found to be the most used part as medicine as they are most easily accessible can also be used in different ways very easily. Different plants and their parts may have a distinct mode of preparations and are preferred over one another according to the user’s flexibility and satisfaction. The simplest and most common modes of preparation are extraction, juice, paste, and decoction. In this study, most people used paste (33%).

Knowledge regarding medicinal plants is obtained from different sources such as family, the local Kaviraaj, and the local people. Consequently, the information gained is discrete, given that, it was found that several plants or plant parts were used for treating the same disease. For instance, for treating the common cold, “Tulsi” (*Ocimum tenuiflorum*), “Bashok” (*Justicia adhatoda*), “Dhan-shabarang” (*Cymbopogan citratus*) etc. were used. Supporting this fact, a study conducted in the Lushai community of Bandarban district of Bangladesh, pointed out that 37 different plant species were used to treat diarrhea and 40 plant species were used to treat dysentery [16]. On the other hand, one particular plant was used to treat many different diseases. The plant “Thankuni” *(Centella asiatica)* was used to treat both diarrhea and gastric. Similarly, “Ghritkumari” (aloe vera) is used as a remedy for both constipation, due to its laxative property which relieves constipation by promoting intestinal motility, as well as diabetes, as aloe vera is believed to improve insulin secretion and enhance pancreatic β cell function [29, 30].

The plant *Heptapleurum hypoleucum* was selected for further study as this has been used most commonly to treat diarrhea in the tribal areas. The stem part of the plant was collected and processed into powder and examined to assay its antibacterial activity against several diarrhea-causing bacteria. From the trials, the highest inhibition zone was seen by ethanolic extract against *Staphylococcus aureus*, which was 21 mm. In contrast, another research work with medicinal plants showed that the chloroform extract of *P. sagitatta* plant showed an inhibition zone of 16 mm against *Staphylococcus aureus* [31]. Comparing the two, the 21 mm inhibition zone from the ethanolic extract seems very significant. However, no zone was observed against *Shigella flexneri* both in methanolic and ethanolic extract. Additionally, the aqueous extract showed results only against *S. aureus* and *S. pneumoniae*. Although the activity for commercial antibiotics is much higher than herbal plants, the use of medicinal plants should be encouraged as the overuse of antibiotics will eventually lead to the development of superbugs which will hinder proper treatment. As the extract of our medicinal plant was not purified and yet we found a comparable value (21 mm in ethanolic extract and 27 mm in commercial antibiotic against *Staphylococcus aureus*), we believe purification of the plant extract will increase its activity significantly.

Medicinal plants certainly contain various types of minerals and both primary and secondary metabolites and for these, they confer antimicrobial effects [32–34]. In this study, phytochemical analysis of the plant extract showed both positive and negative results for several compounds. The plant extract has shown to contain alkaloids, phenolics, steroids, and flavonoids, whereas a negative result was observed for saponins and tannins, which indicates that *Heptapleurum hypoleucum* lack these two phytochemicals.

## Conclusion

Ethnoscience and indigenous knowledge need to be consolidated with modern biotechnological techniques and approaches to achieve the desired end products with scientific validation and to make the most use of the vast amount of natural resources around us. This study suggests that various medicinal plants can serve as an alternative to the mainstream drugs to which bacteria are gradually becoming resistant. From the results, *Heptapleurum hypoleucum* has not only shown significant antibacterial effect against several diarrhea-causing pathogens, but we also found some medically important constituents after conducting the phytochemical tests; thus, further research is recommended to purify these phytochemicals to understand the full potentials of this medicinal plant.

## Data Availability

All data generated or analyzed during this study are included in this published article.

## Abbreviations

MHA: Mueller Hinton Agar
AI: Activity index
GIA: Gastrointestinal ailments
RSD: Respiratory system disorders
PB: Poisonous bites
SMSD: Skeleton muscular system disorders
ED: Endocrinal disorders
HEM: Hemorrhoids
LP: Liver problems
DID: Dermatological infections and diseases
HTN: Hypertension
